# Agreement Between Mucicarmine-Stained Bronchial Brushing and Bronchial Biopsy in Subtyping Non-small Cell Lung Carcinoma

**DOI:** 10.7759/cureus.37848

**Published:** 2023-04-19

**Authors:** Syeda Fatima Rizvi, Shahzada K Sohail, Muhammad Imran

**Affiliations:** 1 Basic Medical Sciences, College of Medicine, University of Bisha, Bisha, SAU; 2 Pathology/Histopathology, Allama Iqbal Medical College, Lahore, PAK

**Keywords:** squamous cell carcinoma (scc), non-small cell lung carcinoma (nsclc), mucicarmine, bronchial brushing, bronchial biopsy, adenocarcinoma lung

## Abstract

Background

Bronchial brushing and biopsy are used for the diagnosis of lung carcinoma as most of these tumors are unresectable. Recently, the subclassification of non-small cell lung carcinoma (NSCLC) into adenocarcinoma (ADC) and squamous cell carcinoma (SCC) has become mandatory due to the emergence of targeted therapies. Due to the inherent limitations of small samples, subcategorization of a tumor is not always possible. Immunohistochemical and mucin stains are being used for this purpose, especially for tumors with poorly differentiated features. In our study, we utilized mucicarmine mucin stain to refine the differentiation of SCC and ADC on bronchial brushing and determine its agreement with bronchial biopsy. This study aimed to measure the degree of agreement between mucicarmine-stained bronchial brushing and bronchial biopsy for subtyping NSCLC into SCC and ADC.

Methodology

This descriptive, cross-sectional study was conducted in the pathology department of Allama Iqbal Medical College. Samples were collected by the pulmonology department of Jinnah Hospital Lahore. The study was conducted for 10 months from June 2020 to April 2021. A total of 60 cases diagnosed as NSCLC, aged between 35 and 80 years, were included in this study. After cytohistological evaluation of bronchial brushing and bronchial biopsy specimens, the agreement was deduced using kappa statistics.

Results

The strength of agreement between mucicarmine-stained bronchial brushing and bronchial biopsy for subtyping NSCLC into SCC and ADC was found to be substantial.

Conclusions

As significant agreement exists between the two modalities, mucicarmine-stained bronchial brushing can be used for a reliable and rapid categorization of NSCLC.

## Introduction

Lung cancer is the most common cause of organ malignancy worldwide [1,2]. Unfortunately, more than two-thirds of lung cancer present at late stages (IIIb or IV), making them unresectable [1,3-5]. Morphological diagnosis is then based on cytology and bronchoscopic biopsy sample [1,3,6].

Earlier the broad subtyping of lung cancer into small cell lung carcinoma (SCLC) and non-small cell lung carcinoma (NSCLC) was considered adequate for treatment purposes [5]. Reportedly, the diagnosis of NSCLC without further specification was preferred in small biopsies to avoid diagnostic discrepancies with subsequent resection specimens [3]. Herein, 30-50% of NSCLCs were not classified further and were designated by the term NSCLC-not otherwise specified (NSCLC-NOS) [3,7]. With the identification of molecular differences and the emergence of target therapy for different subtypes of NSCLC differentiating squamous cell carcinoma (SCC) from adenocarcinoma (ADC) has become necessary [3,5,7,8]. Prediction of the improved response of epidermal growth factor receptor (EGFR)-positive ADC to tyrosine kinase inhibitor and pemetrexed and potential toxicity of bevacizumab in SCC emphasize the need for this differentiation [1-3].

In 2011 International Association for the Study of Lung Cancer/American Thoracic Society/European Respiratory Society proposed separate classification systems, criteria, and terminologies for the diagnosis of small biopsy/cytology and resection specimens [2,3]. The committee incorporated the clinical, radiological, and genetic information in formulating the classification [3]. This proposal for reporting small biopsies and cytology was adopted and incorporated into the latest World Health Organization (WHO) classification system (2015) [9]. The use of histochemical stains and IHC was strongly recommended for subtyping NSCLC, especially those with poorly differentiated features [3]. Using mucin stains and immunohistochemical (IHC) markers the diagnosis of NSCLC-NOS has been reduced from 30-50% to 10% only [7,9].

To save the tissue for necessary molecular testing the use of only one SCC marker (p63 or p40) and one ADC marker (thyroid transcription factor 1, TTF-1) with/without histochemical stain for mucin is recommended [1,3,7-10].

Mucin stains were recommended in 1967 and 1981 WHO classifications of lung cancers, far earlier than the introduction of IHC [3,4]. It still has a vital role, especially in resource-limited settings where IHC is not available [3,9]. Mucin stain has been reported to increase the diagnostic accuracy of poorly differentiated ADC to 88% [7]. Diastase-periodic acid Schiff, Alcian blue, and mucicarmine mucin stains are the most widely used histochemical stains for this purpose [3,4]. Studies showed that for diagnosis of ADC, mucicarmine has similar specificity and higher sensitivity than TTF-1 and Napsin A [4,10].

This study was conducted to determine the agreement between cytological and histological diagnosis in accurately subtyping NSCLC into ADC and SCC with ancillary use of mucicarmine stain on bronchial brushing. If the agreement between the two modalities is strong, mucicarmine-stained bronchial brushings will provide an earlier, cost-effective, and reliable diagnosis to our treating oncologists for decision-making and improving treatment outcomes among patients.

## Materials and methods

This descriptive, cross-sectional study was conducted in the pathology department of Allama Iqbal Medical College, Lahore, over a period of 10 months from June 2020 to April 2021. This research used the same cases that were collected in our previously published study [11] and improvised with the use of mucicarmine stains on bronchial brushing. Ethical approval was obtained from the Institutional Ethical Review Board (ERB) and informed consent was taken from the patients. A total of 60 cases of NSCLC were included in this study using a 95% confidence level and a 12% margin of error, with an expected agreement between bronchial biopsy and mucicarmine-stained brushing as 66% based on the study by Ocque et al. [12]. A non-probability, purposive sampling technique was used. We only included cases that were morphologically diagnosed as NSCLC on both bronchial brushing and biopsy samples. Cases with inadequate cellularity, delayed fixation, not including both types of samples, and diagnosed as other types of cancer were excluded. The required samples for each case were obtained in a single procedure by the pulmonology department of Jinnah Hospital Lahore. The registration numbers of cases were kept the same as in our primary study for the accuracy of conventional bronchial brushing in subtyping NSCLC [11]. They were labeled A for biopsy and B and C for bronchial brushing for routine Papanicolaou (PAP) staining and mucin staining, respectively. Bronchial brushings were stained with PAP and mucicarmine stain (at least one slide of each). Bronchial biopsies were stained with hematoxylin and eosin (H&E) stain using standard protocols. The results of bronchial brush cytology on PAP and mucicarmine stain and the histological findings of the bronchial biopsy were recorded independently. Data were statistically analyzed using SPSS version 27 (IBM Corp., Armonk, NY, USA). The strength of agreement between bronchial brushing cytology with mucicarmine stain and histopathology of bronchoscopic biopsy was calculated based on Landis and Koch benchmarks for evaluating the kappa statistics [13].

## Results

The age range of patients was 35 to 80 years, with a mean age of 56.5 ± 12.04 years. Out of the total 60 cases, 50 (83.3%) were males and the remaining 10 (16.7%) were females, with a male-to-female ratio of 5:1.

**Figure 1:**
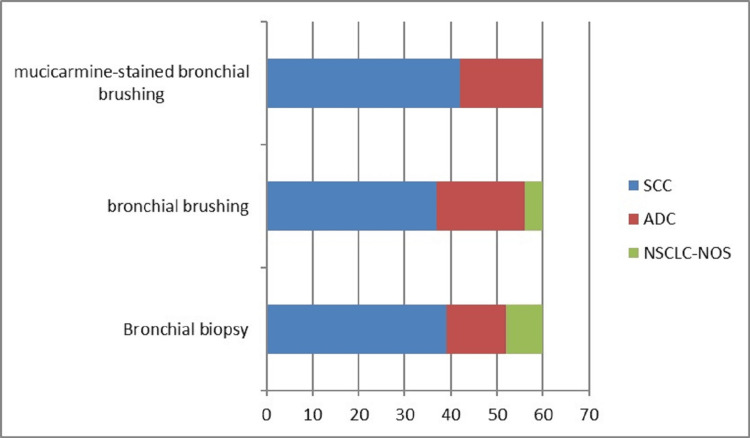
Frequency of the subtype of NSCLC on bronchial biopsy, bronchial brushing, and mucicarmine-stained bronchial brushing. NSCLC: non-small cell lung carcinoma; SCC: squamous cell carcinoma; ADC: adenocarcinoma; NSCLC-NOS: non-small cell lung carcinoma-not otherwise specified

Cytohistological features were used to subclassify all cases into SCC and ADC. The specimens showing characteristic morphological features were diagnosed as SCC (intercellular bridges and keratinization) or ADC (glandular differentiation with mucin production). The tumors without these features were designated as NSCLC-NOS. Figure 1 shows the frequency of ADC, SCC, and NSCLC -NOS as diagnosed on individual specimen types.

The bronchial biopsy and PAP-stained bronchial brushing samples were re-examined by histopathologists, and the diagnosis remained the same as in our previously reported study [11]. On bronchial biopsy, SCC was diagnosed in 39 (65.0%) cases, and ADC was diagnosed in 13 (21.7%) cases. Eight (13.3%) cases could not be subcategorized further and were labeled as NSCLC-NOS [11].

**Figure 2:**
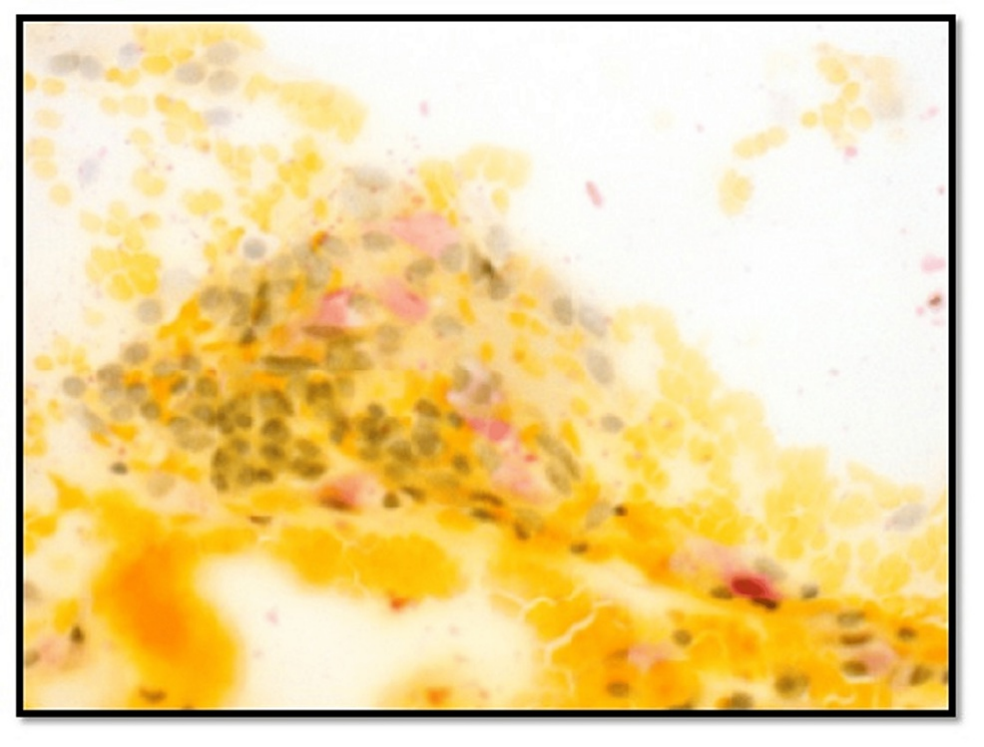
Adenocarcinoma on bronchial brushing (mucicarmine stain, 40×).

On bronchial brush cytology, SCC was diagnosed in 37 (61.7%) cases, and ADC was diagnosed in 19 (31.6%) cases, whereas four (6.7%) cases were labeled as NSCLC-NOS [11]. Mucicarmine-stained bronchial brushing showed 42 (70.0%) cases of SCC and 18 (30.0%) cases of ADC (Figure 2).

The agreement was based on the final diagnosis of a subtype of NSCLC rendered by bronchial biopsy and bronchial brushing with and without the use of mucicarmine stain.

As reported in our primary study, 47 (78.3%) cases showed agreement in the diagnosis of bronchial biopsy and bronchial brushing (Table 1) [11]. Based on the secondary analysis of this finding, the kappa value was determined to be 0.585, taking a 95% confidence interval of 0.414 to 0.755. The number of agreements expected by chance was 28.7 (47.83% of the observations) (Table 2).

**Table 1:** Subtype of NSCLC on bronchial biopsy and brushing. SCC: squamous cell carcinoma; ADC: adenocarcinoma; NSCLC-NOS: non-small cell lung carcinoma-not otherwise specified Reproduced from our published article on the same population [11].

	Diagnosis based on bronchial brushing				Total
Diagnosis based on bronchial biopsy		SCC	ADC	NSCLC-NOS	
	SCC	34	01	04	39
	ADC	00	13	00	13
	NSCLC-NOS	03	05	00	08
Total		37	19	04	60

**Table 2:** Agreement between the subtype of NSCLC diagnosed by bronchial brushing and bronchial biopsy. Symmetric measures based on cross-tabulation (Table 1). a: Not assuming the null hypothesis; b: using the asymptotic standard error assuming the null hypothesis. NSCLC: non-small cell lung carcinoma

		Value	Asymptomatic standard error^a^	Approximate T^b^	Approximate significance
Measure of Agreement	Kappa	0.585	0.087	5.887	0.000
Number of valid cases		60			

The final agreement was based on the diagnosis of the subtype of NSCLC rendered with the bronchial biopsy and mucicarmine-stained bronchial brushing. The diagnostic agreement was observed in 51 (85.0%) cases.

Based on the results, the kappa value was determined to be 0.687, taking a 95% confidence interval of 0.527 to 0.848. The number of agreements expected by chance was 31.2 (52% of the observations) (Tables 3, 4).

**Table 3:** Subtype of NSCLC on mucicarmine-stained bronchial brushing and bronchial biopsy. SCC: squamous cell carcinoma; ADC: adenocarcinoma; NSCLC-NOS: non-small cell lung carcinoma-not otherwise specified

	Diagnosis based on mucicarmine-stained bronchial brushing				Total
Diagnosis based on bronchial biopsy		SCC	ADC	NSCLC-NOS	
	SCC	38	01	00	39
	ADC	00	13	00	13
	NSCLC-NOS	04	04	00	08
Total		42	18	00	60

**Table 4:** Agreement between the subtype of NSCLC diagnosed by mucicarmine-stained bronchial brushing and bronchial biopsy. Symmetric measures based on cross-tabulation (Table 3). a: Not assuming the null hypothesis; b: Using the asymptotic standard error assuming the null hypothesis. NSCLC: non-small cell lung carcinoma

		Value	Asymptomatic standard error^a^	Approximate T^b^	Approximate significance
Measure of agreement	Kappa	0.688	0.082	6.770	0.000
Number of valid cases		60			

The inference drawn for the values is based on the Landis and Koch benchmarks for evaluating the kappa statistics. The strength of agreement between the findings of conventional bronchial brushing and bronchial biopsy was found to be moderate, whereas the strength of agreement between mucicarmine-stained bronchial brushing and bronchial biopsy was found to be substantial.

## Discussion

Most lung cancers are diagnosed at an advanced age, rarely before 40 years of age [2,6,7]. Similarly, our cases showed an age range of 35 to 80 years, with a mean age of 56.5 ± 12.04 years.

The male-to-female ratio was 5:1 in our study, inferring a marked predominance of lung cancer in the male gender. This gender ratio is in accordance with the study by Patel et al. [2].

We included 60 cases of NSCLC and subclassified them based on cytohistological features. On bronchial biopsy, SCC and ADC were diagnosed in 39 (65.0%) and 13 (21.7%) cases, respectively. On bronchial brushing, diagnosis of SCC and ADC was rendered in 37 (61.7%) and 19 (31.6%) cases, respectively. The cases that could not be categorized on cytohistological features were labeled as NSCLC-NOS. Mucicarmine-stained bronchial brushing was diagnosed based on any intracytoplasmic mucin droplets in tumor cells. A total of 42 (70.0%) cases were classified as SCC and 18 (30.0%) cases as ADC. Although ADC surpassed SCC in the West, SCC remains the most frequent type of NSCLC in our population [4,5]. This finding is in concordance with other regional studies [2].

Due to a lack of characteristic morphological features, a number of cases could not be subclassified and were termed NSCLC-NOS. On biopsy, eight (13.3%) cases and on cytology four (6.6%) cases remained uncategorized. In our study, we could subclassify 86.7% of cases into SCC and ADC on biopsy based on morphological features alone. This is in accordance with the findings reported by Kim et al., where they subclassified 78% of their cases based on biopsy samples [7].

The strength of agreement between the findings of bronchial brushing and bronchial biopsy was found to be moderate in this study. This result is in accordance with the studies by Patel et al. and Jain et al., which showed fair-to-high concordance between these modalities in subtyping NSCLC [2,8].

The mucin stain was used as an ancillary technique to facilitate the diagnosis of bronchial brushing. All the cases were reclassified on bronchial brushing based on cytological features combined with the results of mucin stains. Tumors with intracytoplasmic mucin stain were taken as positive and classified as ADC. Mucicarmine-stained bronchial brushing classified 42 (70.0%) cases as SCC and 18 (30.0%) cases as ADC. Based on these results, the strength of agreement between mucicarmine-stained bronchial brushing and bronchial biopsy was found to be substantial. There is limited published data on the use of mucicarmine stain alone; however, it is recommended in combination with the IHC panel to subtyping NSCLC into SCC and ADC [1,3,7]. A study by Micke et al. reported that in the presence of IHC, the value of mucin stains is limited to ambiguous cases only [4]. However, our study targets settings that lack the facility of IHC. In our study, the strength of agreement between the diagnosis of bronchial brushing and bronchial biopsies improved with the ancillary use of mucicarmine stain. This finding agrees with the study by Kim et al., which showed improved diagnostic accuracy with the use of mucicarmine stain [7]. Our study showed that the use of mucicarmine stain will improve the subtyping of NSCLC in poorly differentiated cases; however, more studies are recommended to validate this conclusion.

## Conclusions

The strength of agreement between mucicarmine-stained bronchial brushing and bronchial biopsy was found to be substantial. It implies that the use of mucicarmine stain in conventional cytology smears is highly supportive in the accurate subtyping of NSCLC. An early reliable diagnosis can be provided to the treating physician by use of mucicarmine-stained bronchial brushing for referral of patients for appropriate chemotherapy. This diagnosis can be later verified by bronchial biopsy diagnosis.
